# *NifH*-Harboring Bacterial Community Composition across an Alaskan Permafrost Thaw Gradient

**DOI:** 10.3389/fmicb.2016.01894

**Published:** 2016-11-24

**Authors:** C. Ryan Penton, Caiyun Yang, Liyou Wu, Qiong Wang, Jin Zhang, Feifei Liu, Yujia Qin, Ye Deng, Christopher L. Hemme, Tianling Zheng, Edward A. G. Schuur, James Tiedje, Jizhong Zhou

**Affiliations:** ^1^College of Integrative Sciences and Arts, Arizona State UniversityMesa, AZ, USA; ^2^Arizona State University, Center for Fundamental and Applied Microbiomics, Biodesign InstituteTempe, AZ, USA; ^3^Department of Microbiology and Plant Biology, Institute for Environmental Genomics, University of OklahomaNorman, OK, USA; ^4^Key Lab of the Ministry of Education for Coastal and Wetland Ecosystems, School of Environmental Sciences, Xiamen UniversityXiamen, China; ^5^Center for Microbial Ecology, Michigan State UniversityEast Lansing, MI, USA; ^6^Department of Biological Sciences, Northern Arizona UniversityFlagstaff, AZ, USA; ^7^Earth Sciences Division, Lawrence Berkeley National LaboratoryBerkeley, CA, USA; ^8^State Key Joint Laboratory of Environment Simulation and Pollution Control, School of Environment, Tsinghua UniversityBeijing, China

**Keywords:** *nifH*, nitrogen-fixing, permafrost, diazotroph, nitrogen, microbial

## Abstract

Since nitrogen (N) is often limiting in permafrost soils, we investigated the N_2_-fixing genetic potential and the inferred taxa harboring those genes by sequencing *nifH* gene fragments in samples taken along a permafrost thaw gradient in an Alaskan boreal soil. Samples from minimally, moderately and extensively thawed sites were taken to a depth of 79 cm to encompass zones above and below the depth of the water table. *NifH* reads were translated with frameshift correction and 112,476 sequences were clustered at 5% amino acid dissimilarity resulting in 1,631 OTUs. Sample depth in relation to water table depth was correlated to differences in the *NifH* sequence classes with those most closely related to group I *nifH*-harboring Alpha- and Beta-Proteobacteria in higher abundance above water table depth while those related to group III *nifH*-harboring Delta Proteobacteria more abundant below. The most dominant below water table depth *NifH* sequences, comprising 1/3 of the total, were distantly related to *Verrucomicrobia*-*Opitutaceae*. Overall, these results suggest that permafrost thaw alters the class-level composition of N_2_-fixing communities in the thawed soil layers and that this distinction corresponds to the depth of the water table. These *nifH* data were also compared to *nifH* sequences obtained from a study at an Alaskan taiga site, and to those of other geographically distant, non-permafrost sites. The two Alaska sites were differentiated largely by changes in relative abundances of the same OTUs, whereas the non-Alaska sites were differentiated by the lack of many Alaskan OTUs, and the presence of unique halophilic, sulfate- and iron-reducing taxa in the Alaska sites.

## Introduction

Permafrost underlies 24% of the northern hemisphere land surface and contains approximately 1672 Pg C (Schuur et al., 2008; Tarnocai et al., 2009). Thawing of permafrost from increasing temperatures results in more carbon becoming available to microbial decomposition and hence elevates ecosystem respiration of CO_2_ (Hobbie et al., 2000; Mack et al., 2004; Dutta et al., 2006). Nitrogen is a common limiting nutrient such that N-availability may play an important role in regulating plant productivity as well as microbial decomposition and hence the release of greenhouse gasses CO_2_ and N_2_O. In permafrost, the assimilation of organic N by boreal and Arctic plant species indicates the overall scarcity of inorganic N (Kielland, 1994; Persson et al., 2003). In Alaskan permafrost, long-term N-addition has also been shown to increase decomposition rates, thereby reducing ecosystem C storage (Keller et al., 2010).

The majority of microbial metabolic activity in permafrost is relegated to the active layer that thaws seasonally. Extended thawing periods result in an increase in the depth of this layer, with water flowing through the deeper soil (Keller et al., 2010) which may introduce N from the mineral soils into the upper thawed soil layers (Keuper et al., 2012). Experimental warming of boreal (taiga) and tundra soils supports this, with an observed increased soil inorganic N (Schimel et al., 2004; Natali et al., 2011). Deeper thawing below the rooting zone contributes to the export of inorganic N from the soil to groundwater and/or surface water, especially in discontinuous permafrost zones (Harms and Jones, 2012), thereby bypassing the shallow organic soil where N removal through denitrification and N-retention by assimilation occurs.

Biological N-fixation is an important source of N to Arctic terrestrial systems (Barsdate and Alexander, 1975; Chapin and Bledsoe, 1992; Hobara et al., 2006) since atmospheric deposition of N alone cannot account for excess net organic N uptake rates (Harms and Jones, 2012). Among the soil properties, moisture plays a large role in the variation of N-fixation among Arctic vegetative communities (Chapin, 1996; Stewart et al., 2011) with higher rates occurring under elevated temperatures and moisture (Liengen and Olsen, 1997; Zielke et al., 2005; Stewart et al., 2012). Higher N-availability from enhanced N-fixation in Arctic ecosystems may further increase N-mineralization rates (Giblin et al., 1991; Chu and Grogan, 2010) and hence nitrification rates (Robertson and Vitousek, 1981; Norton and Stark, 2011), resulting in additional NO_3_^-^ available for denitrification and thus the potential for increased N_2_O emissions (Chapin, 1996; Kool et al., 2011). However, net ecosystem gain of N may be reduced as a result of the balance between N-fixation and denitrification (Chapin, 1996; Sorensen et al., 2006), the later being favored by the wet soils, although low denitrification rates have been observed in some Arctic environments (Buckeridge et al., 2009). Ultimately, increased N-availability may also inhibit N-fixation through negative feedback once N-availability surpasses a threshold concentration. Overall, the relationship of N-fixation to soil respiration is unclear as correlations between N-fixation and CO_2_ production vary across ecosystem types (Stewart et al., 2012).

The diversity of the N-fixing genes and their host microbial populations can be assessed by sequencing of the *nifH* gene, which encodes a subunit of the nitrogenase complex whose diversity (Hsu and Buckley, 2009) and abundances (Reed et al., 2010) have been associated with N-fixation rates. Five primary groups of genes homologous to *nifH* have been identified (Young, 1992, 2005; Raymond et al., 2004). Group I consists of aerobic N_2_-fixing bacteria composed of a mix of Alpha-, Beta-, and Gamma-Proteobacteria, among others. Group II are very similar in function and structure to group I and contain mostly obligate anaerobes such as methanogens, sulfate-reducers and clostridia. Group III consists of anaerobic N_2_-fixers from *Bacteria* and *Archaea* and contains primarily Delta Proteobacteria while groups IV and V contain *nifH* paralogs that do not fix nitrogen (Raymond et al., 2004). Conceptually, changes in microbial N-fixation rates can reflect: (i) an alteration in cell-specific activities that may reflect the environment, (ii) changes in abundance of N-fixing organisms, or (iii) a shift in the community composition of N fixers. The aim of this study was to assess differences in the composition of the N_2_-fixing genes and the inferred microbes harboring those genes along a long-term permafrost thaw gradient and in soil above and below the water table In addition, we compared the composition of the *nifH*-harboring bacterial community between two Alaska sites to other sites from diverse ecosystems to determine the shared and habitat unique OTUs.

## Materials and Methods

### Site Description

Soils cores were collected from three sites representing a thaw gradient of minimally, moderately and extensively thawed conditions within the discontinuous permafrost zone from the Eight Mile Lake (EML) watershed, west of Healy, Alaska, USA (63°52′42.1′′N, 149°15′12′′W, 700 m.a.s.l) (Schuur et al., 2007, 2009; Vogel et al., 2009; Lee et al., 2010; Trucco et al., 2012). Permafrost degradation has been ongoing at this region over the past 50 years as a result of regional climate change in Alaska (Schuur et al., 2009; Osterkamp et al., 2009). Spatially random processes that occur at a local level as a series of positive feedbacks between temperature change, ground subsidence, hydrologic redistribution, and further thawing from thermal erosion due to moving water have created the observed gradient from minimal to extensive permafrost thaw at the plot level where samples are collected.

Six cores were taken from each site to a maximum depth of 116 cm and sliced in five to eight sections, depending on the depth of the organic and mineral layers. The minimally thawed site is characterized by the least permafrost degradation and has moist acidic tundra vegetation comprised of graminoid sedges, dwarf evergreen and deciduous shrubs, and forbs with an understory of non-vascular mosses and lichens (Schuur et al., 2007). The moderately thawed site exhibits some subsidence and a deeper active (seasonally thawed) layer, with the initiation of thawing observed in 1990. Plant community composition remained similar to the minimal thaw site, but overall the plants were more productive (Schuur et al., 2007). The extensively thawed site has the deepest active layer with thaw initiation thought to have occurred several decades prior to that at the moderately thawed site (Lee et al., 2010). Vegetation at the extensive site had shifted away from graminoids toward a dominance by shrubs and forbs. In addition, hydrophilic moss species were more prevalent where the water table was at the soil surface as a result of ground subsidence. Re-distribution of surface water caused by this subsidence was observed at the moderately thawed site and was widespread at the extensively thawed site. Volumetric water content at 10 cm ranged from 0.1 to 0.3 ml cm^-3^ while at 30 cm it was near 1.0 ml cm^-3^ at all sites. Depth of the thawed layer at the time of sampling in May was 15.6 ± 0.4 cm, 16.6 ± 0.25 cm, and 26.0 ± 2.3 cm for the minimally, moderately and extensively thawed sites, respectively (Lee et al., 2010). Depth to the water table from six adjacent sites from June to September ranged from 9.9 to 28.9 cm below the soil surface, averaging -17.4 ± 4.1 cm. (Supplementary Figure S1). Soils from below the water table were characterized by redox mottling, though this was not directly quantified. A total of 25 samples composed of ∼10 cm core slices were denoted as above water table (AWT) depth with a maximum penetration of -22 cm into the soil (average -15.4 ± 5.7 cm) while a total of 53 samples comprised the below water table (BWT) samples and averaged -45.4 ± 14.0 cm in depth (Supplementary Figure S1). Organic C pools to 1 m depth averaged 59.8 ± 2.8 kg C m^-2^ at all sites (Schuur et al., 2009). Total aboveground plant biomass has been reported to be 361, 488, and 486 g m^-2^ with mean summer soil temperatures at 10 cm at 8.7, 10.2, and 11.3°C in the minimally, moderately and extensively thawed sites, respectively (Schuur et al., 2007). Soil pH values from sites located ∼2 km from this study site are approximately 4.7, 4.8, and 5.1 for the 0–15 cm, 15–25 cm, and 45–55 cm depths. Total soil C and N reported in this study were measured on core subsamples using a Costech ECS 4010 Elemental Analyzer (Valencia, CA, USA).

### Amplification and Sequence Processing

Primers PolF/PolR tagged with multiplex identifier (MID) sequences were used for the amplification of *nifH* sequences (Poly et al., 2001b) with the Roche Titanium adapter A on PolF and adapter B on PolR. Triplicate 20 μl PCR reactions and a control were performed and reactions purified using the protocol described by Wang et al. (2013). Sequencing was performed using the Roche 454 (GS-FLX Titanium) platform by Macrogen (Geumcheon-gu, Korea).

Nucleotide sequences were processed through the Ribosomal Database Project (RDP) pyrosequencing pipeline^1^. Chimeric sequences were identified and removed using UCHIME6.0 running in *de novo* mode. Sequences were translated to protein and frameshift-corrected using the RDP’s protein-coding gene tool (FrameBot; Wang et al., 2013) and *nifH* database. Protein reads were re-sampled to 1,000 sequences per sample using R (R Core Team, 2013) followed by alignment and complete linkage clustering at 5% amino acid dissimilarity. Sequences pertaining to representative sequences as the minimum sum of the square of the distances within each cluster were generated. A hand-curated *nifH* database (augmented Zehr-set; Wang et al., 2013) with the extracted protein region corresponding to that amplified by the primers was used as the reference set for BLASTp analyses. Sequences were deposited in the European Nucleotide Archive under study accession PRJEB7072, secondary accession ERP006756, and sample accessions ERS533154–ERS533209.

Raw reads were Hellinger-transformed (square root of relative abundance) and Bray–Curtis (+1) dissimilarity matrices constructed for community statistical analyses using the PRIMER-6 software package (Clarke and Warwick, 2001). Treatment differences were tested by analysis of similarity (ANOSIM) (Clarke, 1993), permutational analysis of variance (PERMANOVA) (Anderson, 2001), Non-metric dimensional scaling (NMDS) and similarity percentages (SIMPER) (Warwick et al., 1990), and permutational dispersion (PERMDISP) (Anderson et al., 2006). The Chao estimate of community richness was used for *nifH* diversity estimates. Aligned amino acid sequences were trimmed and neighbor joining trees were constructed using MEGA6 (Tamura et al., 2013) and visualized in iTOL (Letunic and Bork, 2011). Samples were grouped according to site (minimally–moderately–maximally thawed), depth below surface and depth relative to the water table depth. Amino acid sequences were obtained from Wang et al. (2013) for comparison of the *nifH*-harboring microbial communities among sites. ANOVA statistics were carried out using Minitab^®^16. Samples within 5-cm below the average annual water table depth were included, since some 10-cm core slices included up to 5-cm below this value but were also within the range of water table variation (Supplementary Figure S1).

## Results

### *NifH* Diversity Analyses

A total of 124,703 raw sequences were recovered from 78 samples, from which 112,476 sequences remained with an average length of 344 bases after quality filtering and removal of 29 samples that contained less than 1,000 reads. After removal of chimeric sequences, all remaining samples were randomly re-sampled to 1,000 sequences for further analyses. A total of 1,631 OTUs (549 singletons) were generated after clustering at 5% amino acid identity.

**FIGURE 1:**
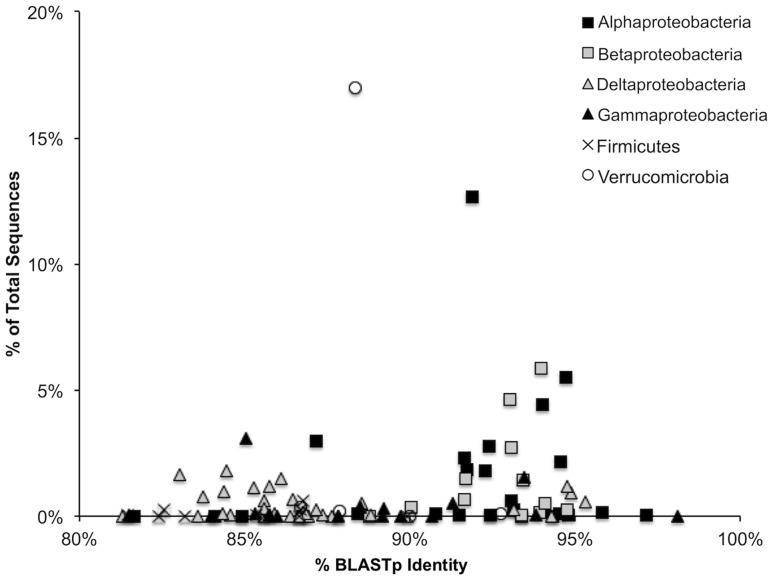
**Percent BLASTp identity of representative sequences within each of the represented bacterial groups against a hand-curated nifH database.** A total of 95.7% of all sequences are represented.

Average protein-protein identity to the curated database was 89.4 ± 4.8% at an average alignment length of 106 amino acids. Percent identity to the reference database was highest for the Alpha- and Beta-Proteobacteria (**Figure 1**). Protein sequences corresponded to those of 123 unique closest match species/strains comprising 79 genera and 11 phyla in the reference database. The top 15 closest match genera accounted for 86.0% of all sequences and 73.1% of all OTUs.

A total of 74.1% of sequences were most closely matched to the Proteobacteria and the majority of high abundance OTUs were closest to the order Rhizobiales such as *Methylobacterium nodulans* (12.7% of all sequences), *Rhizobium* sp. (6.7%), *M. silvestris* (5.5%), and *Bradyrhizobium* sp. (7.9%). Outside of the Proteobacteria, closest matches were to the *Verrucomicrobia*, specifically hits to *Opitutaceae* sp. TAV2 (16.4%) were the most abundant. *Desulfovibrio*-related sequences were the most diverse, with 5.2% of all sequences in 8.9% of all OTUs. In contrast, *Methylobacterium*-related sequences were the least diverse, with 12.7% of all sequences in 2.7% of all OTUs. Percent nitrogen and carbon were obtained for a portion of the BWT samples only and were not found to be significantly different along the thaw gradient (ANOVA) (Supplementary Table S1).

### Changes in Diazotrophic Communities with Depth

**FIGURE 2:**
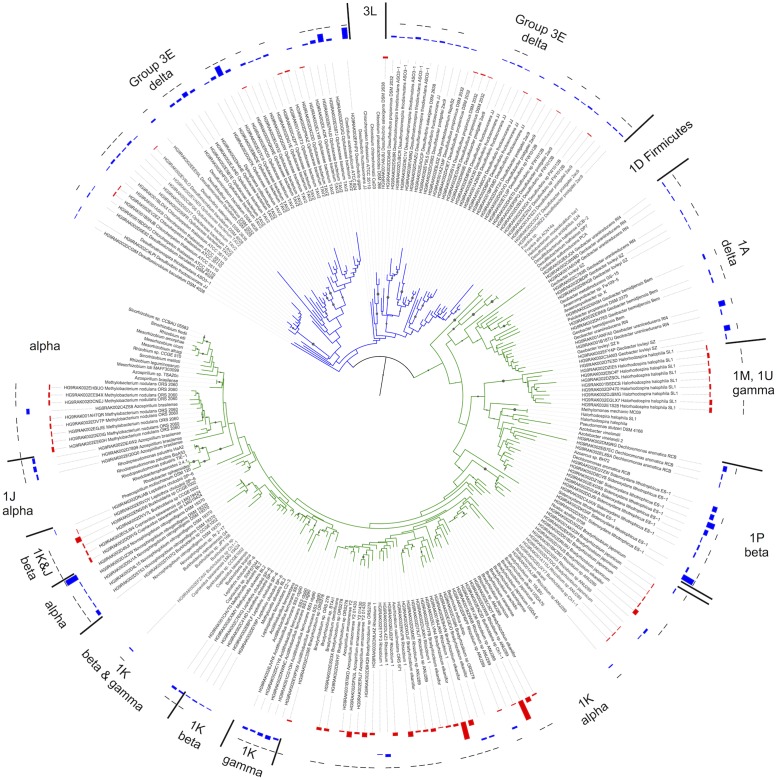
**Neighbor joining tree based on 116 comparable NifH amino acid positions using reference sequences obtained from the hand-curated NifH database used for BLASTp assignment.** Cluster representative sequences representing those clusters that constituted the SIMPER results were chosen. Bootstrap values >50% are indicated by branch symbols. Group names (e.g., 3E) indicated are based upon the ARB database. The innermost two data fields indicate the relative abundances for each OTU in the above water table (AWT-red) and below water table (BWT-blue). The outermost field indicates significant differences (*t*-test, *p* < 0.05) between the BWT and AWT relative abundances (black bar = significant, no bar = not significant).

Significant changes in the composition of the diazotroph community were found based on position above (AWT) and below (BWT) the water table (PERMANOVA; *F* = 2.19 *P* < 0.001, ANOSIM; *P* < 0.001), roughly corresponding to the thaw depth at time of sampling. Chao richness estimators were significantly higher in the BWT samples versus AWT (ANOVA; *F* = 4.78, *P* = 0.03) (Supplementary Figure S2). A neighbor-joining tree was constructed to visualize the differences in relative abundances between the AWT and BWT layers (**Figure 2**). Two distinct clades of the group III and one clade of group 1A *nifH*-harboring taxa consisting of Delta Proteobacteria were most associated with the deeper layers while group I (especially 1 K) containing Alpha Proteobacteria were higher in abundance in the samples above the water table. *NifH* sequences associated with the Delta Proteobacteria were clearly separated from the other groups. Tree branch length differences were smaller between the Alpha-, Beta-, and Gamma-Proteobacteria that may affect the confidence in assigning finer taxonomic resolution.

**FIGURE 3:**
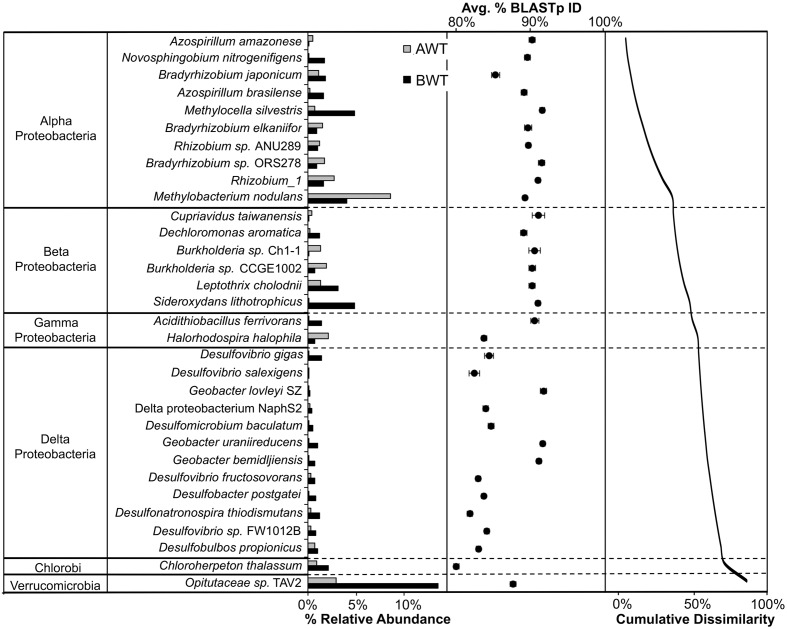
**Similarity Percentage (SIMPER) analysis showing the representative sequence BLASTp closest match identity of the OTUs that contribute significantly to the discrimination between above water table (AWT) and BWT *nifH*-harboring bacterial communities.** The OTUs shown contribute 79.1% of the cumulative dissimilarity, shown in the second pane. Average percent BLASTp identity with standard errors are shown in the third pane. These taxa account for 25.8% of all closest match reference taxa.

**FIGURE 4:**
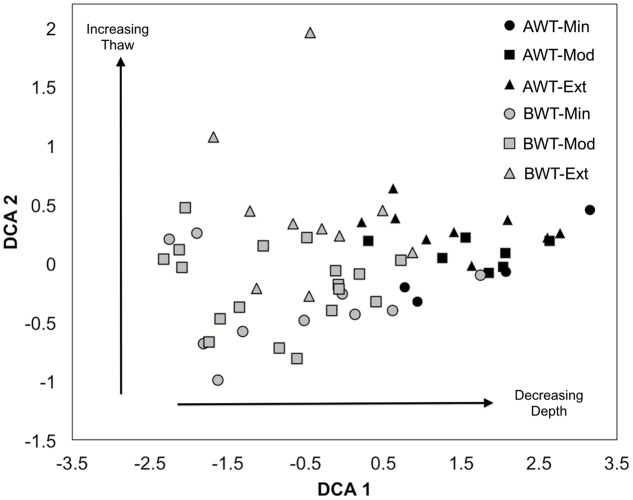
**De-trended correspondence analysis ordination of *nifH*-harboring bacterial communities in all cores related to thaw depth.** AWT = above water table, BWT = below water table, Min = minimally thawed site, Mod = moderately thawed site, Ext = extensively thawed site. Arrows indicate trends with depth (AWT versus BWT: PERMANOVA, *F* = 2.19 *P* < 0.001) and with increasing/decreasing thaw status (Min, Mod, Ext: PERMANOVA, *F* = 0.89 *P* = 0.53). Differences between minimal versus extensively thawed sites were the most highly contrasted (PERMANOVA pairwise comparison, *P* = 0.06). Multivariate dispersion indices (MVDISP) were 0.88 for BWT and 1.44 for AWT and tested with permutational dispersion (PERMDISP; *F* = 10.40 *P* < 0.01).

The contribution of individual closest-match genera to the overall Bray–Curtis distances (SIMPER analysis) showed that differences in the abundances of 32 OTUs accounted for 79.1% of the discrimination between the AWT and BWT soil layers (**Figure 3**). These 32 OTUs are derived from a subset of those OTUs shown in **Figure 2**. The sequences most related to Alpha Proteobacteria (within group I) were more associated with the AWT layers with those most closely matching Delta Proteobacteria (group III) having larger populations in the BWT layers. This is most pronounced with the abundance of sequences in BWT most closely related to the *Verrucomicrobia Opitutaceae* sp. TAV2 (*Diplosphaera colitermitum* TAV2). Delta Proteobacterial sequences from the BWT layer had lower BLASTp percent identities than the majority of sequences associated with the AWT layer. The BWT layer contained 19 OTUs unique to that layer (15.4% of all OTUs, 0.04% of all sequences) while the AWT layer only contained four unique OTUs containing <0.01% of all sequences. The overall diazotrophic community structure was largely shaped by position above or below the average water table, with higher variation between samples observed in the deeper soil samples (**Figure 4**).

### Changes in nifH Communities Along the Thaw Gradient

Overall there was no significant change in nifH-harboring bacterial community structure with thaw extent (PERMANOVA; *F* = 0.89 *P* = 0.53, ANOSIM; *P* = 0.12) according to the position of the soil core along the gradient (minimally–moderately–and extensively thawed sites). A total of five closest match taxa (3756 sequences) significantly (*t*-test, *p* < 0.05) changed between the minimally and extensively thawed sites when all depths were considered. These changes were the result of relative abundance increases in the extensively thawed site with closest BLASTp matches related to *Desulfovibrio* that increased 8-fold, *Bradyrhizobium* (2x), *Methylocella* (6x), *Zymomonas* (5x), and *Desulfobulbos* (4x). *NifH* derived Chao richness did not significantly change across the thaw gradient.

### Comparison of Two Alaskan Sites

**FIGURE 5:**
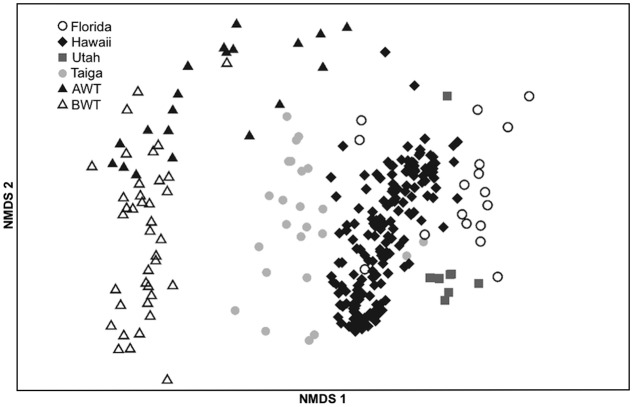
**NMDS ordination of *nifH* harboring bacterial communities from this study (AWT = above water table, BWT = below water table) and those obtained from the NEON study (Wang et al., 2013) corresponding to samples taken in Florida, Hawaii, Utah and a Taiga forest. 2D stress = 0.12**.

To investigate whether there is a “core” Alaskan *nifH*-harboring bacterial community, we combined our results with a study that used identical sequencing and processing methodologies with soils located in an Alaskan forested taiga (Wang et al., 2013), also above permafrost, located 1390 km from the thaw gradient, and three very different ecosystems. NMDS ordination (**Figure 5**) illustrates the significant (PERMANOVA, *F* = 36.24, *p* < 0.001; PERMDISP, *p* = 0.49) differences between all sites. The average PERMANOVA-derived similarities of replicates within the sites were similar (65.3 ± 1.8%) while the Alaska AWT samples were most similar to the BWT samples (54.3%) followed closely by the Alaskan taiga soils (51.9%), Hawaii (44.2%), Florida (41.9%), and Utah (41.5%). However, overall the taiga samples were more similar to the Hawaii, Florida, and Utah samples than to our AWT or BWT samples.

Since position above or below the average water table depth was shown to contribute largely to community differences, we then compared the AWT data to the AK taiga for further analyses. A total of 72 of 83 closest match taxa at >0.1% abundance were shared and relative abundances for OTUs present in both datasets were highly correlated (Spearman rank correlation, ρ = 0.995). Among these, the closest match *M. nodulans* ORS2060, a nodulating symbiont, was abundant at both sites (17.9%-AWT, 8.2%-forested taiga) as were those most similar to the genera *Rhizobium* (18.3% vs. 9.3%), *Bradyrhizobium* (5.6% vs. 13.0%), *Halorhodospira* (9.8% vs. 5.7%), and *Leptothrix* (2.9% vs. 3.9%) (Supplementary Figure S3). A total of 26 of the 83 taxa at >0.1% abundance significantly (*t*-test, *p* < 0.05) changed between AK sites. The more abundant significantly changing taxa included closest matches to *Azospirillum* (1.0%-AWT, 6.8%-taiga), *Burkholderia* (4.8% vs. 2.3%), *Sideroxydans* (0.1% vs. 5.2%), *Rubrivax* (3.0% vs. 0.1%), and *Anaeromyxobacter* (0.0% vs. 2.6%), among others.

## Discussion

We examined *nifH* gene diversity along a natural thaw gradient and established that water table depth was a significant contributor to shaping the *nifH*-harboring bacterial community structure. The recovered alpha diversity was based on 1,631 OTUs from our 95% amino-acid dissimilarity clustering. However, due to inherent biases, directly comparing studies on *nifH* diversity as well as all other amplified functional genes can be problematic due to varying bioinformatic methods (Penton et al., 2013). These biases have been documented among the most widely adopted *nifH* primers (Gaby and Buckley, 2012), including PolF/PolR, used here, which preferentially target the Proteobacteria (Penton et al., 2013; Wang et al., 2013). Regardless, Mao et al. (2011) analyzed *nifH* diversity using 697 sequences per sample, also using PolF/PolR primers. They identified 229 OTUs at 90% amino acid dissimilarity in 21 samples, lower than our 1,631 OTUs from 60 samples at 95% dissimilarity. In another study, 31 of the 45 *nifH*–inferred cultivable genera that were shared among four sites in South and North America (Roesch et al., 2010) were identified in our samples. Our *NifH* richness estimates, which were error-corrected by FrameBot, indicate high *nifH*-harboring bacterial diversity in our samples. In comparison, Duc et al. (2009) estimated a species richness of 20 near a receding alpine glacier while others (Poly et al., 2001a; Rösch and Bothe, 2005; Deslippe and Egger, 2006) identified lower richness but with the more limited sampling due to use of cloning methodology.

A hand-curated reference protein database was utilized containing 675 *NifH* protein sequences in order to assign OTU representative sequences to a nearest match. These nearest-neighbor assignments are made without regard to the percent identity between the query and target sequence and without a specific cutoff threshold. A study utilizing *nifH*-harboring taxa that included 16S rRNA gene sequences determined that *NifH* amino acid distances varied widely within phylogenetic clusters; 41.7% among methanogens, 12.8% among Alpha, and 12.9% among Gamma Proteobacteria (Zehr et al., 2003). Other comparisons of nucleotide identities revealed that 80% of strains that shared 97% 16S rRNA identity had <95% *nifH* identity, 43% of those had less than 85% *nifH* nucleotide identity and a set of strains with 99% 16S rRNA gene identity corresponded to ∼77% *nifH* identity (Gaby and Buckley, 2012). Therefore the use of a universal identity cutoff threshold when assigning to a nearest neighbor is not directly supported. This also complicates the choice of an appropriate clustering dissimilarity value, as diversity will likely be over- or underestimated in some bacterial groups.

Horizontal gene transfer (HGT) must also be considered when assigning taxonomy to functional gene sequences. Recent transfer events have not been identified though there is evidence of ancient HGT between groups I and III (Gaby and Buckley, 2012). While based on a smaller subset of taxa, Zehr et al. (2003) also found a lack of evidence supporting recent HGT. Our analyses show that the group III sequences are more deeply diverging than group I, indicative of the lower group I substitution rates found by Gaby and Buckley (2012). As such, the differentiation of group I versus group III based on *nifH* sequences appears possible. In addition, taxonomic assignment of group III *nifH*-harboring taxa is supported due to higher sequence divergence. However, taxonomic differentiation of sequences within group I may be problematic due to the comparatively small divergences and incongruent phylogenetic relationships.

**FIGURE 6:**
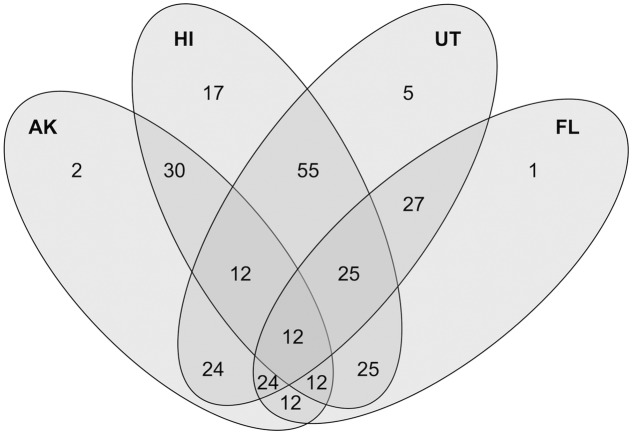
**Venn Diagram showing the shared and unique closest match taxa of 286 total OTUs between the combined above water table (AWT) and taiga samples (combined as AK), Hawaii (HI), Utah (UT), and Florida (FL) with the criteria that a certain closest-match taxa is present in at least 50% of all samples in a site**.

The majority of differences between the AK and Florida, Utah, and Hawaii sites were not due to changes in relative abundances, but rather to absence in the AK sites of specific OTUs (**Figure 6**). Taxa most associated with AK included several halophiles, aquatic species and those associated with respiration of alternate electron acceptors, reflecting ecosystem pressures on the proliferation or the exclusion of certain lineages. Halophiles have been found to be a feature of permafrost samples (Steven et al., 2007), due to the distribution of brine channel habitats. Comparisons between our AK AWT samples and the AK taiga site revealed that presence-absence drove differences between these more similar habitats within the rarer (<0.1%) members while the more abundant OTUs varied in relative abundances.

### Water Table Influences

Whether the core originated in the minimally, moderately or extensively thawed site had relatively little impact on the overall composition of the diazotrophic community. Instead, the largest influence was the position of the sample above or below the water table. Significantly higher Chao richness in the BWT layers and differences in *nifH*-harboring bacterial community structure is likely a reflection of the influence of anaerobiosis due to water-saturated conditions and/or the composition of the bacterial community before or during permafrost formation in the Pleistocene epoch. Once thawed and above the standing water table, as reflected in the AWT layers, the bacterial community shifts to populations selected by higher redox conditions that appear influenced to a greater extent by vegetation. Long-term increases in thaw depth will result in deeper penetration of the aerobic layer as additional water produced during permafrost thaw is transported, transpired or evaporated. While depth was a significant correlate to community change, it appears that temperature increases at a 10 cm depth along the gradient (8.7°C -minimally thawed to 11.3°C -extensively thawed) (Schuur et al., 2007) did not further impact bacterial community structure, with no significant change in the *nifH*-harboring bacterial community in the AWT layers.

Differences between the AWT and BWT diazotrophic communities were not attributed to presence/absence but were rather due to changes in relative abundances. The deep, separate branching of the group III Delta Proteobacteria sequences from the group I *nifH*-containing Alpha- and Beta-Proteobacteria supports the contrasts made between the AWT and BWT layers. However, genus-level closest match taxonomic assignments reported in this study, particularly in the Alpha- and Beta-Proteobacteria, may not be robust. With a few exceptions, the OTUs most closely matching the group I Alpha- and Beta-Proteobacteria were mostly associated with the AWT layer, where plant roots are more likely to reside and enrich the diazotrophic community such as the group 1 k clade that includes *Bradyrhizobium*, *Rhizobium*, and the *Burkholderia*, The consistently larger abundances of OTUs related to group III *nifH*-harboring Delta Proteobacteria in the BWT layer are likely linked to the lower redox state in the deeper sediments supporting these sulfate (e.g., *Desulfovibrio*) and Fe(III)-reducing (e.g., *Geobacter*) *nifH*-harboring taxa. However, with the exception of the *Geobacter* species, the remaining dominant closest-similarity taxa of the BWT *nifH* community, compared to the AWT, exhibited lower % similarity to the closest reference match. This is especially true with the group III *Desulfovibrio*, whose average amino acid similarity of 85.5 ± 0.2% was lower than the average within-genus reference set variability of 94.5 ± 1.5%, but close to the nearest-neighbor protein similarity of *D. gigas* (85.9%), suggesting that they may be uncultured *Desulfovibrio* species. In addition, *M. nodulans* 2060 is the only known N-fixing *Methylobacterium*. This organism was identified as the closest reference for 12.7% of all protein sequences and present in higher abundance in the AWT layers. However, their uncertain phylogenetic placement in the tree (**Figure 2**) and lack of a known plant host (leguminous plants of the genus *Crotalaria*) suggests that these reads are likely uncultured soil bacteria, as previously suggested in other AK samples (Wang et al., 2013). Overall, these low amino acid identities are likely due to the retrieval of potentially novel organisms from a combination of sampling the less well-understood permafrost ecosystem and the maximum soil sampling depth of 90 cm.

The depth at which a phylogenetic shift of the diazotrophic community occurs was at the water table interface that corresponded to thaw depth at the time of sampling. The largest specific difference between depths was attributed to OTUs assigned to the group III *Opitutaceae* sp. TAV2 (*D. colitermitum*). The TAV2 isolate is a *Verrucomicrobia* and originally isolated from the hindgut of a termite, *Reticulitermes flavipes* (Stevenson et al., 2004). It is microaerophilic, possesses the *nifHDK* genes as well as a functional nitrogenase (Wertz et al., 2012). The closest database relative to *Opitutaceae* sp. TAV2 is another *Verrucomicrobia* bacterium DG1235 at 93.5% a.a. identity, suggesting that a currently uncultured *Verrucomicrobia* may exert a significant impact on N-availability, as proposed for *Verrucomicrobia* previously in acidic and oligotrophic ecosystems (Wertz et al., 2012). In addition, this OTU may be related to the large population bin of *Verrucomicrobia*–*Opitutus* sp. identified in soil metagenomes from a closely related site (Johnston et al., 2016). The larger heterogeneity observed in the deeper sample replicates (**Figure 4**) likely illustrates the spatially constrained evolution of divergent communities due to the frozen state, and thus, low mixing in the deeper layers of the permafrost.

Community shifts have also been observed at the active layer/permafrost boundary where permafrost metagenomes were found to functionally (but not phylogenetically) converge toward active layer metagenomes during a short-term thaw experiment (Mackelprang et al., 2011). Q-PCR based *nifH* abundances decreased with thaw in that study while *nifH*, total bacteria and fungi were found to increase in abundance in another study (Yergeau et al., 2010). At an International Tundra Experiment (ITEX) site, *nifH* gene diversity decreased with experimental warming (Walker et al., 2008) in concert with significantly higher N-fixation rates in the surface samples (Henry and Svoboda, 1986). While taxonomic information or quantitation alone cannot be used to predict N-fixation rates, the phylogenetic shift toward more putative plant-associated taxa in the thawed layers suggests a functional linkage with the aboveground biomass.

Of the multiple edaphic factors that play a role in plant community composition, N-availability has a particularly strong influence in tundra systems, changing species composition and enhancing growth (Shaver et al., 2001; Mack et al., 2004; Walker et al., 2006). As a proxy for increasing active layer depth, a significant change in plant composition has also been identified between our minimally and extensively thawed sites with a shift from graminoid to shrub dominance with significantly higher total green tissue N. This was interpreted to be a result in an increase in the N supply rate from the soil (Schuur et al., 2007) from either enhanced decomposition or N_2_-fixation. Indeed, N-fertilization resulted in a similar graminoid to shrub conversion with a corresponding increase in productivity and decomposition but without a change in active layer depth in another study (Mack et al., 2004). The combination of these findings in concert with our data suggests that increases in thaw depth that correspond to a shallower water table depth may initiate changes in aboveground plant community composition through a combination of edaphic factors that subsequently influence the diazotroph community. The question remains as to whether these community changes can be explicitly linked to higher N-fixation rates that potentially drive enhanced decomposition, higher soil N supply and plant green tissue N. Conversely, higher N-fixation may increase denitrification rates as the water table rises with a concomitant increase in N_2_O emissions. However, the establishment of a direct linkage between changes in diazotrophic community composition and potential outcomes in this permafrost system hinges on the magnitude of changes in N-fixation rates. Furthermore, this is dependent on whether changes in cell (or taxa) specific N-fixation rates accompany the observed community turnover, if other environmental properties ultimately control these rates and the balance between denitrification and N-fixation. In order to establish these explicit linkages *in-situ* measurements of N-fixation, nitrification and denitrification are needed to establish a N-mass balance of the system. In addition, a greater understanding of taxa-specific N-fixation rates is required to link diazotroph population dynamics to changes in ecosystem level N-availability.

## Author Contributions

CP performed data analysis and wrote the manuscript. CY conceived this aspect of the project and performed laboratory work. YD, FL, CH, JZ, and YQ performed laboratory work and processed sequencing data. LW processed and cataloged samples and oversaw laboratory operations. QW performed bioinformatic analysis and provided tools for sequence processing. ES collected the samples and maintained the Alaskan study site. JT, JZ, TZ, and ES were involved in the organization, planning and management of the overall project. JT, ES, and JZ were involved in peer-editing of the manuscript.

## Conflict of Interest Statement

The authors declare that the research was conducted in the absence of any commercial or financial relationships that could be construed as a potential conflict of interest.

## Footnotes

1 http://rdp.cme.msu.edu
