# Supplementary figures and images for: NifH-Harboring Bacterial Community Composition across an Alaskan Permafrost Thaw Gradient
Source: Front Microbiol. 2016 Nov 24;7:1894. doi: 10.3389/fmicb.2016.01894 (PMC5121533; doi:10.3389/fmicb.2016.01894)

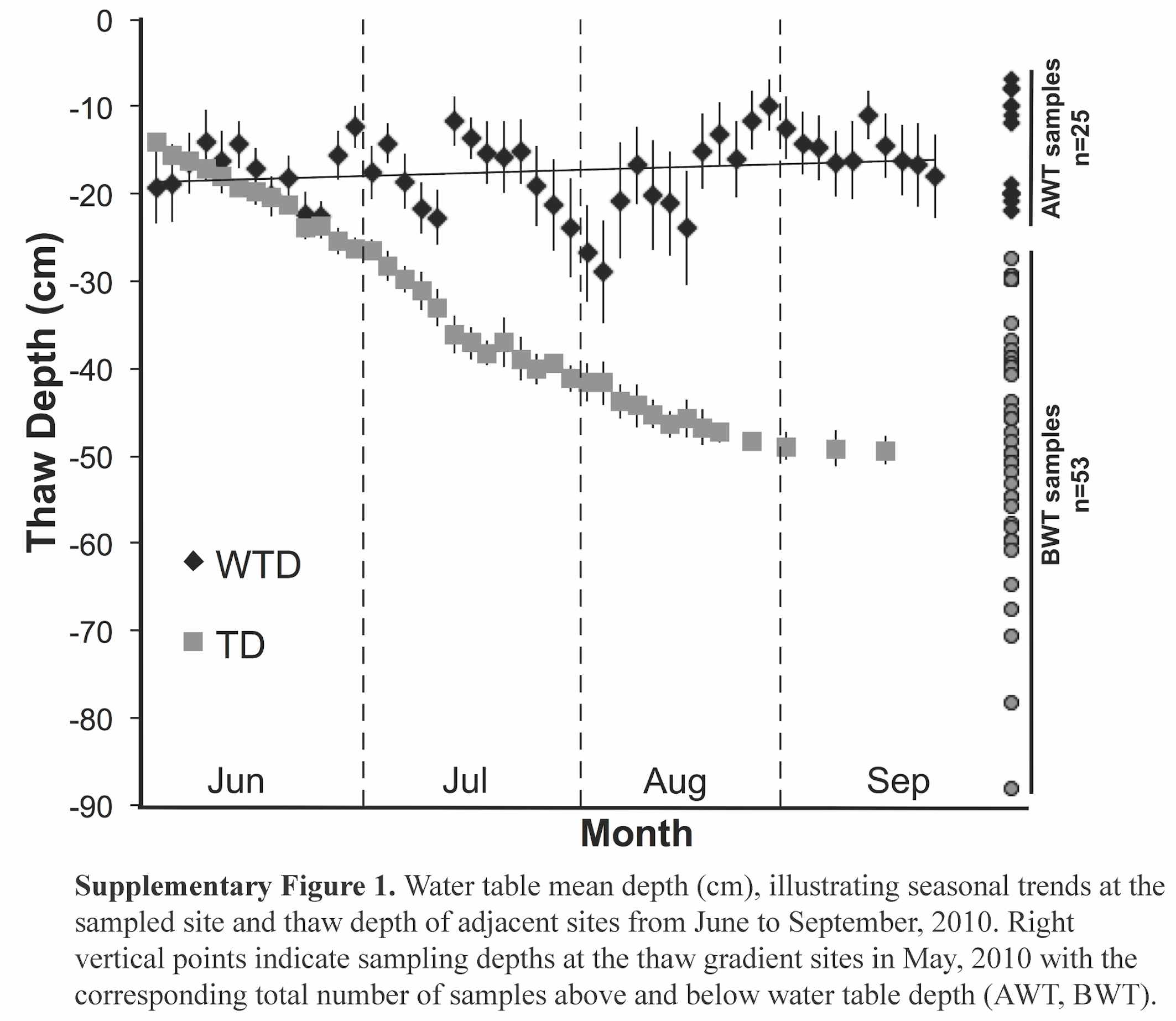

Supplement: Supplementary file 1 [file Image_1.TIF]

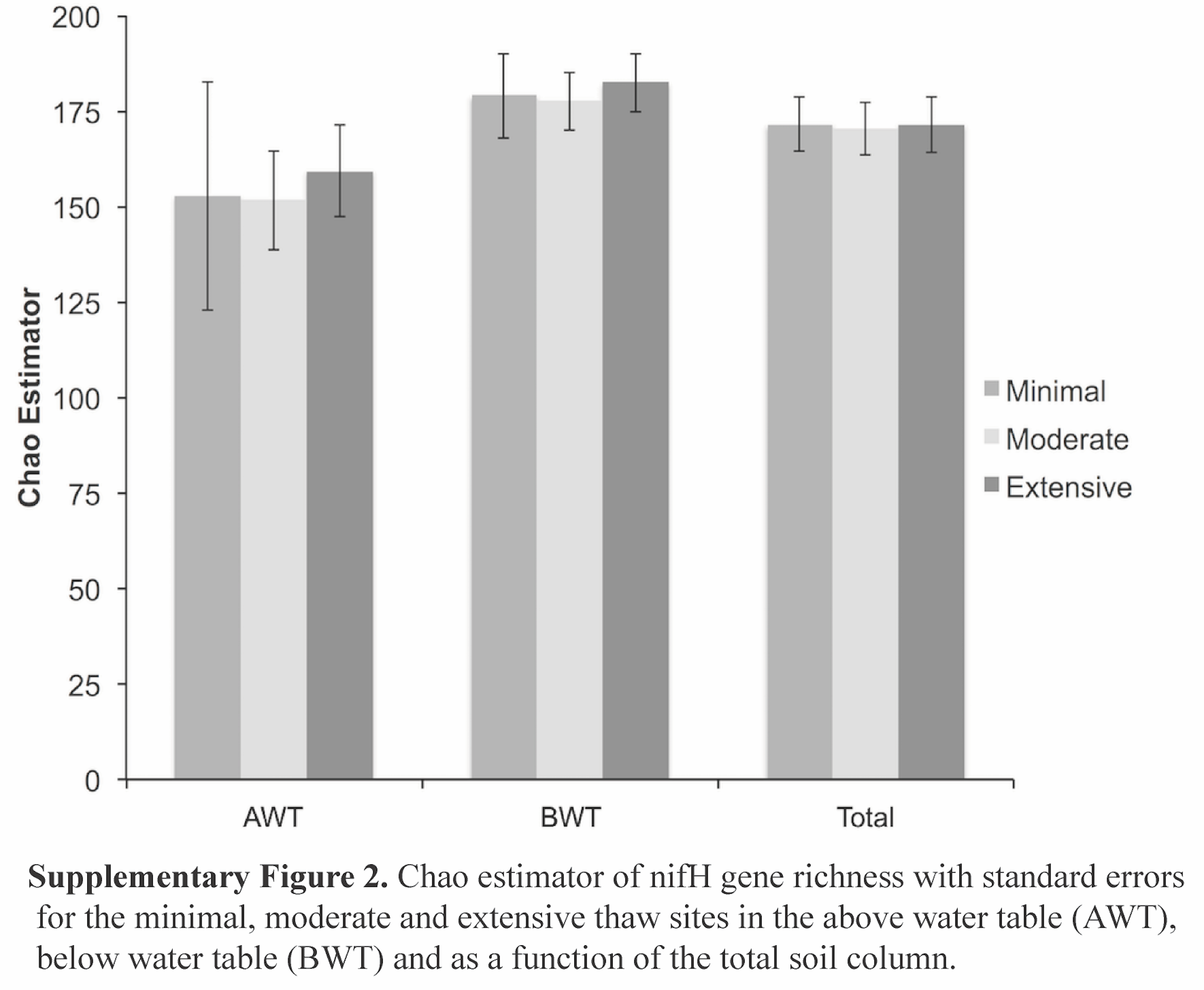

Supplement: Supplementary file 2 [file Image_2.TIF]

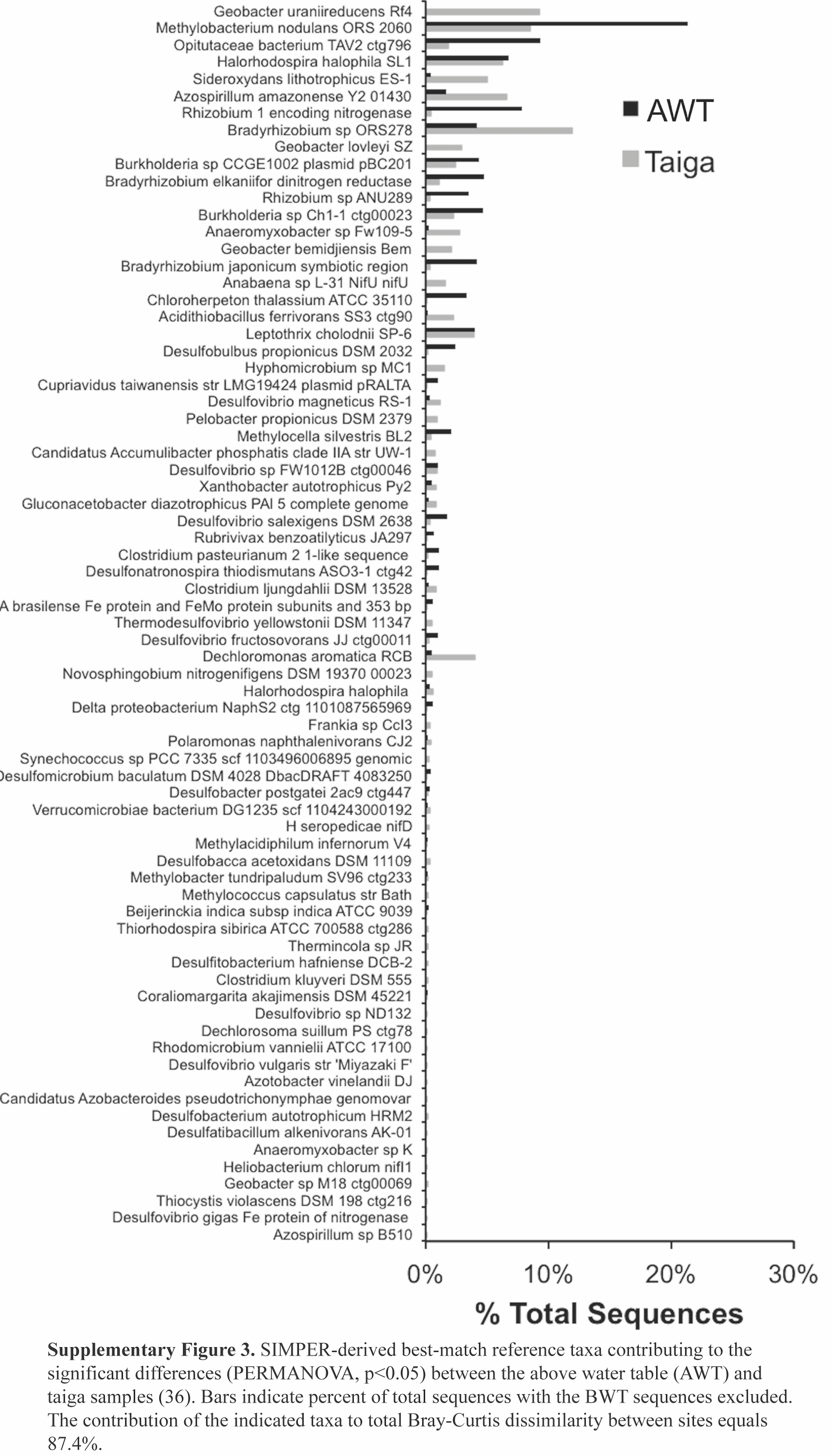

Supplement: Supplementary file 3 [file Image_3.TIF]
