# Supplementary material for: NifH-Harboring Bacterial Community Composition across an Alaskan Permafrost Thaw Gradient
Source: Front Microbiol. 2016 Nov 24;7:1894. doi: 10.3389/fmicb.2016.01894 (PMC5121533; doi:10.3389/fmicb.2016.01894)
Supplement: Supplementary file 4 [file Table_1.PDF]

Supplementary Table 1. Average percent nitrogen and carbon in above (AWT) and below (BWT) water table samples from the extensive, moderately and minimally thawed sites with standard errors and ANOVA grouping with Tukey corrections.

Layer	Thaw Status	% N	% C
AWT	Extensively	1.11 ± 0.11 ^{A}	36.92 ± 3.05 ^{A}
	Moderately	0.83 ± 0.13 ^{A}	41.71 ± 0.55 ^{A}
	Minimally	0.99 ± 0.19 ^{A}	41.88 ± 1.16 ^{A}
BWT	Extensively	1.32 ± 0.07 ^{A}	32.87 ± 2.02 ^{A}
	Moderately	1.18 ± 0.12 ^{A}	27.29 ± 2.58 ^{A}
	Minimally	1.11 ± 0.13 ^{A}	27.81 ± 2.80 ^{A}
